# Factors Influencing the Thermo-Hydro-Mechanical Behavior of Unstabilized Rammed Earth Walls

**DOI:** 10.3390/ma15248821

**Published:** 2022-12-10

**Authors:** Xiang Zhang, Hossein Nowamooz

**Affiliations:** ICube, UMR 7357, CNRS, INSA de Strasbourg, 24 Boulevard de la Victoire, 67084 Strasbourg, France

**Keywords:** unstabilized rammed earth (URE) materials, environmental solicitations, rising damp, time effect, thermal-hydro-mechanical coupling, numerical simulations

## Abstract

Waterproof capacity, thermal isolation, and pushover strength are the main characteristics when an unstabilized rammed earth (URE) wall is constructed. In this paper, a comprehensive numerical simulation model is built to evaluate the effect of 15 different factors on those three aforementioned properties of URE walls. The simulation results show that the hydraulic, thermal, and mechanical properties of the wall are interconnected. It is found that the waterproof capacity of the wall can be mainly improved by increasing the dry density, decreasing the rising damp effect, and reducing the fine content value of the wall. The thermal insulation characteristic of the wall can be ameliorated by increasing the wall thickness and reducing the rising damp effect, fine content, and dry density. In addition, the pushover capacity of the wall can be strengthened by increasing the wall width, fine content, wall thickness, and vertical load and decreasing the rising dampness and wall height. In addition, time has a positive effect on the waterproof capacity, thermal insulation, and mechanical strength of URE walls. These properties change significantly in the first 100 days and then stabilize after 180 days for a typical URE wall. Eventually, a new theoretical approach is proposed to predict the long-term THM behavior of URE walls by considering the 15 factors in its framework.

## 1. Introduction

In recent years, unstabilized rammed earth (URE) has gained popularity as an economical and environmentally friendly material that is typically compacted with local soil in a formwork using a pneumatic or manual rammer [1,2]. However, the properties of URE are poorly understood when rammed earth is considered a contemporary building technique. Some related codes and standards emphasize geometric relationships without a comprehensive understanding of the material’s properties [3]. Various factors, such as compaction energy [4], soil type [5], and optimum water content [6], influence the material properties of URE from the beginning of the construction process onward. Therefore, to understand the characteristics of URE, factors influencing its performance must be identified during the design stage.

The first major factor is the initial state of unstabilized rammed earth (URE) material. URE is generally compacted with a dry density between 1750 kg·m^−3^ to 2000 kg·m^−3^ and gravimetric water content in the range of 4% to 13% [3]. The compaction of soil materials (maximum dry density) decreases with the saturation state and the optimum water content [7]. Therefore, the material properties can be different due to the different compaction conditions. Raavi and Tripura [8] showed that the compressive strength of rammed earth increases with dry density. Similar experimental results were also observed by Wangmo et al. [9] and Lin et al. [10]. Johari et al. [11,12] found that nano-clay is an environmentally friendly material that can be used to improve the bearing capacity of URE materials. Luo et al. [13] found that, due to the effect of rainfall, a higher initial water content leads to a higher degree of erosion and a lower degree of erosion is observed when the clay content is higher. Rammed earth materials have poor frost resistance under high water content, especially at the early age stage and under rising damp conditions [14].

Once the unstabilized rammed earth (URE) material is compacted, the moisture conditions of the material start changing over time [15]. Water then flows in or out through the material surface due to wetter or dryer ambient conditions. Then, the THM properties consequently vary due to the variation of the water content [16,17]. When the water content decreases, the compressive strength, tensile strength, and elastic modulus of URE increase [18]. François et al. [19] simulated rammed earth constructions under hygroscopic conditions, where their model showed that the water permeability of URE increases with water content. Fabbri et al. [20] found that the vapor permeability of URE decreases when the water content increases. Chabriac et al. [21] observed that the volumetric water content in the URE wall decreases with time due to gravity and evaporation effects. Ávila et al. [3] concluded that the thermal conductivity of URE increases with the increase of dry density and water content.

Another crucial factor affecting masonry buildings is the effect of rising dampness and evaporation [22] caused, respectively, by the humid state of ground soil and ambient air. Rising water leads to bad indoor conditions, poor thermal insulation, and material deterioration [23]. On the contrary, water loss due to evaporation inhibits progress and improves masonry structure properties [24]. Some authors [17,19,25] considered the interactions of unstabilized rammed earth (URE) and air conditions; however, the effect of rising damp has rarely been investigated experimentally or numerically in the literature. Jiang et al. [17] observed variable relative humidity inside the URE wall due to the different humidity on both sides of the wall. Saneiyan and Slater [26] found that water increases the electrical conductivity of soil, and Abdulsamad et al. [27] provided a method using electrical conductivity to image the change in water content of URE walls. Dong et al. [25] observed that the thermal performance of rammed earth structures varies with ambient air conditions.

Another factor is the dimensions of the unstabilized rammed earth (URE) wall. El Nabouch [28] observed that the pushover strength of the URE wall increases when the wall height decreases. Serrano et al. [29] found that the reduction of wall thickness worsens the thermal insulation of rammed earth. Torres [24] found that rising dampness effects are enhanced by the increase in wall thickness.

In addition, some other factors influencing the properties of soil and masonry structures have been considered for unstabilized rammed earth (URE) materials. Talev et al. [30] tested the convective moisture transfer coefficient for different porous building materials, and the results show that the evaporation process is enhanced by the increase of wind velocity and air temperature. Zhang et al. [31] concluded that the increase in wind speed increases the heat loss process of the exterior wall surface. An et al. [32] expressed the heat source of radiation as the combination of shortwave radiation and longwave radiation (affected by cover [33]) for the soil–atmosphere interactions. Nowak [34] pointed out that the longwave radiation incident upon a building should also be considered for building structures. In addition, the net shortwave radiation is influenced by the albedo value which is affected by soil colors [35].

As mentioned above, despite a large number of investigations on the factors influencing the thermo-hydro-mechanical (THM) behavior of earth material performance, most existing studies focused only on the effect of a limited number of factors. In addition, there is no available numerical study considering the field conditions for rammed earth walls thoroughly and considering the effect of several factors simultaneously. Moreover, most experimental results were obtained at an equilibrium stage where the effect of time is seldom considered. Furthermore, seldom studies focused on establishing a model that can be applied to predict the coupled long-term THM behaviors of the unstabilized rammed earth (URE) structure under in situ conditions.

In this paper, we investigate the periodical variation of waterproof capacity, thermal insulation, and pushover strength of unstabilized rammed earth (URE) walls by considering the effects of material type, compaction state, outdoor relative humidity, outdoor temperature, wall thickness, wall height, wall width, outdoor wind speed, cloud cover, incoming shortwave radiation, and vertical load. After that, an analytical approach is proposed to predict the coupled long-term THM behaviors of unstablized rammed earth (URE) walls under in situ conditions.

## 2. Governing Equations of the Numerical Framework

This section provides a summary of the governing equations for numerical simulations of unstabilized rammed earth (URE) walls, taking into account heat transfer, water flow, and yield surfaces.

### 2.1. Water Balance through the Wall Surface

Water in the wall evaporates from the surface of the wall in the form of water vapor:

(1) $$-n\cdot v_{w}\rho_{w}=\frac{h_{m}M_{w}}{RT}\left(RH_{e}p_{sat}-RH_{i}p_{sat}\right),$$

where h_m_ (m·s^−1^) is the moisture transfer coefficient, and RH_e_ and RH_i_ are the external and internal relative humidity on the surface, respectively.

h_m_ with no wind speed (v_wind_) is calculated according to Tong et al. [36], and when the wind speed is not zero, h_m_ is obtained using the moisture transfer coefficient of pure water under the assumption that only the water phase is affected:

(2) $$h_{m}=\{\begin{array}{c} 0.01885668C_{wi0}\frac{R\sqrt{T}}{M_{w}},v_{wind}=0\\ \frac{nD_{v}Sh}{L},v_{wind}>0 \end{array},\;$$

where C_wi0_ is a material-dependent coefficient taken equal to 4.4 × 10^−6^, Sh (-) is the Sherwood number, D_v_ (m^2^·s^−1^) is the vapor diffusivity, and L (m) is the length of the evaporative surface taken equal to 0.3 m [37].

The wind speed changes with different height levels and the wind speed on the surface of the wall built in a city can be calculated through Hellmann’s exponential law [38,39,40].

(3) $$v_{wind}=v_{wind0}\cdot {\left(\frac{H}{H_{0}}\right)}^{\alpha_{H}},$$

where v_wind0_ (m·s^−1^) is the speed obtained from the average wind speed value at the height H_0_ (m). H_0_ is frequently referred to as 10 m, H (m) is the height of the wall, and α_H_ (-) is the Hellman exponent taken as 0.4 for the buildings in city residential areas.

The water vapor diffusivity is obtained through [41]

(4) $$D_{v}=2.12\times 10^{-5}{\left(\frac{T}{273.15}\right)}^{2}.$$

The Sherwood number is calculated through [42]:

(5) $$Sh=0.145Re^{0.69}Sc^{0.87},\;$$

where Sc(-) is the Schmidt number and

(6) $$Sc=\frac{\mu_{v}}{D_{v}\rho_{v}},\;$$

where μ_v_ (Pa·s) is the dynamic viscosity of vapor.

The speed of vapor and wind is assumed to be the same on the boundary, and the Reynolds number Re (-) is then a dimensionless number that depends on air properties:

(7) $$Re=\frac{v_{wind}\rho_{v}L}{\mu_{v}}.\;$$

### 2.2. Energy Balance through the Wall Surface

The energy balance through the wall surface is determined as follows:

(8) $$R_{n}+H_{s}-H_{l}-S=0,\;$$

where R_n_ (W·m^−2^) is the net radiation heat flux, H_s_ (W·m^−2^) is the sensible heat flux, H_l_ (W·m^−2^) is the latent heat flux, and S (W·m^−2^) is the surface heat flux. It should be noted that net radiation and sensitive heat flux both have positive and negative values, while latent heat flux only has positive values. Surface heat flux indicates the total heat flux flow through the wall surface. To calculate the surface heat flux, it is necessary to obtain the net radiation heat flux, the sensitive heat flux, and the latent heat flux.

At first, the net radiation on the wall surface is the combination of the solar shortwave, longwave radiation, and the longwave radiation of the ground:

(9) $$R_{n}=\left(1-a_{lb}\right)R_{s}+\left(R_{Lg}+R_{La}-\varepsilon_{e}\sigma_{B}{\left(T_{ext}+273.15\right)}^{4}\right),\;$$

where a_lb_ (-) is the surface albedo, R_s_ (W·m^−2^) is the local global shortwave radiation, R_La_ (W·m^−2^) is the incoming longwave radiation of the environment, R_Lg_ (W·m^−2^) is the incoming ground longwave radiation, ε_e_ (-) is the emissivity of the wall, σ_B_ (W·m^−2^·K^−4^) is the Stefan–Boltzmann constant, and T_ext_ (K) is the outdoor temperature of the wall.

The shortwave radiation is influenced by the albedo value of the wall, which is taken as a function of the Meter Munsell color value [35]:

(10) $$a_{lb}=0.069c_{cv}-0.114,\;$$

where c_cv_ (-) is the Meter Munsell color value of the wall.

The environmental longwave radiation is affected by the cloud cover [34,43,44]:

(11) $$R_{La}=\left(\begin{array}{c} 5.61^{-13}{\left(T_{ext}+273.15\right)}^{6}\cdot \left(1-C_{c}\right)+\left(5.61^{-13}{\left(T_{ext}+273.15\right)}^{6}+69.3C_{c}\right)\cdot C_{c} \end{array}\right)\cdot K_{1}\;+K_{3}\cdot \left(0.09\left(1-\left(0.9203+0.0043T_{ext}\right)\cdot C_{c}\right)\right)\cdot \sigma_{B}\cdot {\left(T_{ext}+273.15\right)}^{4},\;$$

in which C_c_ (-) is the cloud cover factor, and K_1_ (-) and K_3_ (-) are the parameters dependent on the inclination angle of a wall taken, respectively, as 0.5 and 0.3457.

The ground longwave radiation R_Lg_ incident upon the building surface is obtained through [34]

(12) $$R_{Lg}=\left(159.5+2.77T_{ext}\right)sin^{2}\left(\beta_{inc}/2\right),\;$$

where β_inc_ is the wall inclination angle equal to π/2.

Secondly, the sensible heat flux is provided as the convective heat transfer between the wall surface and the ambient air:

(13) $$H_{s}=h_{eff}\left(T_{e}-T_{i}\right),\;$$

where h_eff_ (W·m^−2^·K^−1^) is the effective heat transfer coefficient, and T_e_ (K) and T_i_ (K) are, respectively, the external and internal temperature on the wall surface.

The effective heat transfer coefficient is affected by wind speed and is provided by [31]

(14) $$h_{eff}=-0.0203v_{wind}^{2}+1.766v_{wind}+12.263.$$

Thirdly, the latent heat flux through the wall surface is obtained by consulting the water flux:

(15) $$H_{l}=-n\cdot v_{w}\rho_{w}L_{v}.\;$$

L_v_ (J·kg^−1^) is the latent heat of evaporation. The latent heat of evaporation L_v_ is calculated by [17]

(16) $$L_{v}=\left(2500-2.4\left(T-273.15\right)\right)\times 10^{3}.\;$$

### 2.3. Heat Transfer in the Wall

The following equations based on Fourier’s law are then used to determine heat transfer where the heat caused by water vapor condensation and material deformation is neglected:

(17) $$\rho_{eff}C_{eff}\frac{\partial T}{\partial t}=\rho_{v}C_{v}v_{v}\cdot \nabla T+\rho_{w}C_{w}v_{w}\cdot \nabla T-\nabla \left(\lambda_{eff}\nabla T\right),\;$$

where ρ_eff_ (kg·m^−3^) is the effective density of URE materials, C_eff_ (J·kg^−1^·K^−1^) is the effective specific heat capacity of the material, T (K) is the temperature, t (s) is time, ρ_v_ (kg·m^−3^) is the density of water vapor, C_v_ (J·kg^−1^·K^−1^) is the specific heat capacity of water vapor, v_v_ (m·s^−1^) is the velocity of water vapor which is assumed equal to the velocity of liquid water, C_w_ (J·kg^−1^·K^−1^) is the specific heat capacity of water, and λ_eff_ (W·m^−1^·K^−1^) is the effective thermal conductivity of the material.

The density of water vapor is calculated as follows:

(18) $$\rho_{v}=\frac{M_{w}p_{sat}RH}{RT},\;$$

where M_w_ (kg·mol^−1^) is the molar mass of water molecules, p_sat_ (Pa) is saturated water vapor pressure, R (J·K^−1^·mol^−1^) is the gas constant, and RH (-) is the relative humidity.

The saturated vapor pressure p_sat_ is calculated as follows [45]:

(19) $$p_{sat}=\frac{exp\left(34.494-\frac{4924.99}{237.1+T}\right)}{{\left(T+105\right)}^{1.57}}.\;$$

The specific heat capacity of water vapor C_v_ is calculated according to ref. [46], and the density of liquid water ρ_w_ is calculated as follows [47]:

(20) $$\rho_{w}=-0.0228{\left(T-273.15\right)}^{2}-0.1176\left(T-273.15\right)+999.9.$$

The specific heat capacity of water C_w_ is provided by [48]

(21) $$C_{w}=8958.9-40.535T+0.11243T^{2}-1.014\times 10^{-4}T^{3}.\;$$

The effective density ρ_eff_ (kg·m^−3^) and the effective specific heat capacity C_eff_ (J·kg^−1^·K^−1^) are presented as [49]

(22) $$\rho_{eff}=\left(1-n\right)\rho_{s}+n\left(\left(1-S_{r}\right)\rho_{v}+S_{r}\rho_{w}\right),\;$$

(23) ρCeffeff=ρsCs(1−n)+n((1−Sr)ρvCv+SrρwCw), 

where n (-) is the porosity, ρ_s_ (kg·m^−3^) is the soil particle density, which is equal to 2600, S_r_ (-) is the saturation, and C_s_ (J·kg^−1^·K^−1^) is the specific heat capacity of particles.

An average value of specific heat capacity of RE particles C_s_ equals 1081 J·kg^−1^·K^−1^ is used in this study according to the experimental results reported in the literature [17,50,51,52,53,54]. The effective thermal conductivity λ_eff_ (W·m^−1^·K^−1^) increases with dry density and saturation, the effects of dry density [17,50,51,52,53,54,55,56,57,58,59,60] and saturation [17,50,51,52,57,58] are investigated, and all the experimental results regarding effective thermal conductivity are calibrated with the approach proposed by refs. [61,62]:

(24) $$\lambda_{eff}=0.031\gamma_{d}\cdot \frac{2Sr}{1+Sr}+0.044\gamma_{d},\;$$

where γ_d_ (kN·m^−3^) is the dry unit weight of URE.

In the building industry, the RSI value (K· m^2^ ·W^−1^) is used to measure the wall resistance to the heat flow. The RSI value is calculated as follows:

(25) $$RSI=\frac{L_{th}}{\lambda_{eff}},\;$$

where L_th_ (m) is the thickness of the wall.

### 2.4. Water Flow in the Wall

The water flow in porous media [63,64,65] is generally written as follows:

(26) $$\nabla \left(nS_{r}D_{w}\right)\nabla \rho_{w}-\frac{S_{r}\rho_{w}}{E}\frac{\partial s}{\partial t}-\frac{nC_{m}}{g}\frac{\partial s}{\partial t}+nS_{r}\frac{\partial \rho_{w}}{\partial t}+\nabla \rho_{w}v_{w}+\left(nS_{r}\rho_{w}\right)\frac{\partial \varepsilon_{v}}{\partial t}=0,\;$$

where D_w_ (m^2^·s^−1^) is the diffusivity of water (considered 0 in this study due to its negligible effect), s (Pa) is the total suction, E (Pa) is the elastic modulus at failure, C_m_ (m^−1^) is the specific liquid water capacity, and ε_v_ (-) is the volumetric strain.

The total suction is calculated through the Kelvin equation [66] by relative humidity and temperature variation:

(27) $$s=-\frac{\rho_{w}RT}{M_{w}}lnRH.\;$$

The liquid water velocity is calculated through the generalized Darcy’s law [67]:

(28) $$v_{w}=-\frac{\kappa \kappa_{rw}}{\mu_{w}}\nabla \left(s+\rho_{w}gD\right),\;$$

where κ (m^2^) is the saturated water liquid water permeability, κ_rw_ (-) is the relative liquid water permeability, μ_w_ (Pa·s) is the dynamic viscosity of liquid water, g (m·s^−2^) is the gravity acceleration, and D (m) is the water head.

The dynamic viscosity of liquid water is obtained as follows [68]:

(29) $$\mu_{w}=5.0\times 10^{-6}{\left(T-273.15\right)}^{2}-3.0\times 10^{-5}\left(T-273.15\right)+0.0018$$.

The saturated liquid water permeability is obtained through the Kozeny–Carman equation [69,70]:

(30) $$\kappa =C_{KC}\cdot \frac{e_{s}^{3}}{1+e_{s}},\;$$

where C_KC_ (m^2^) is a material constant parameter equal to 3.81 × 10^−13^ [20,71] in this study, and e_s_ (-) is the void ratio of the material.

The effects of drying–wetting cycles on the soil water retention curve (SWRC) are neglected in this study, and the saturation is obtained through the van Genuchten (VG) [72] model:

(31) $$S_{r}=\{\begin{array}{cc} \frac{1}{{\left(1-{\left(\alpha_{VG}\frac{s}{\rho_{w}g}\right)}^{n_{VG}}\right)}^{m_{VG}}} & ,s>0\\ 1 & ,s<0 \end{array}.\;$$

The constant parameters m_VG_ (-) and n_VG_ (-) are equal to 1.4 and 0.29 in this study. α_VG_ is inversely related to the air entry value [72]. α_VG_ (m^−1^) is correlated to the fine content (c_fi_) of the URE material as follows:

(32) $$\alpha_{VG}=0.01\cdot c_{fi}^{-1}.\;$$

Moreover, the relative liquid water permeability is calculated through the Mualem equation [73]:

(33) $$\kappa_{rw}=\{\begin{array}{cc} S_{r}^{l_{VG}}{\left(1-{\left(1-S_{r}^{\frac{1}{m_{VG}}}\right)}^{m_{VG}}\right)}^{2} & ,s>0\\ 1 & ,s<0 \end{array},\;$$

where l_VG_ (-) is a constant material parameter equal to 0.5 [33,74].

The specific liquid water capacity is calculated as follows [72]:

(34) $$C_{m}=\{\begin{array}{cc} \frac{\alpha_{VG}m_{VG}}{1-m_{VG}}\left(\theta_{s}-\theta_{r}\right)S_{r}^{\frac{1}{m_{VG}}}{\left(1-S_{r}^{\frac{1}{m_{VG}}}\right)}^{m_{VG}} & ,s>0\\ 0 & ,s<0 \end{array},\;$$

where θ_r_ (-) is the residual volumetric water content of the studied material, its value is considered a constant value of 0.0045, and θ_s_ (-) is the saturated volumetric water content.

The volumetric water content θ (-) is equal to the porosity of the saturated state. For unsaturated conditions, the volumetric water content is obtained through the following expression: θ = θ_r_ + S_r_∗(θ_s_ − θ_r_).

### 2.5. Mechanical Equations

The mechanical equilibrium equations are provided by Newton’s second law:

(35) $$\rho_{eff}\frac{\partial^{2}u}{\partial t^{2}}=\nabla \sigma +f_{v},\;$$

where **u** is the displacement vector, **σ** is the Cauchy stress tensor, and **f_v_** is a body force per unit deformed volume.

According to Hooke’s law, the stress increment is described as

(36) $$d\sigma_{ij}=D_{ijkl}^{el}d\varepsilon_{kl}^{el},\;$$

where dσ_ij_ is the stress increment, $D_{ijkl}^{el}$ is the elastic modulus tensor obtained using the Poisson coefficient λ (-), and the elastic modulus at failure E (Pa). $d\varepsilon_{kl}^{el}$ is the elastic strain increment. The Poisson coefficient λ is considered constant and equal to 0.25 [19].

The material strains are calculated as follows:

(37) $$\varepsilon_{ij}=\varepsilon_{ij}^{el}+\varepsilon_{ij}^{pl}=\frac{1}{2}\left(\frac{\partial u_{i}}{\partial x_{j}}+\frac{\partial u_{j}}{\partial x_{i}}\right),\;$$

where ε_ij_ (-) is the engineering strain tensor, $\varepsilon_{ij}^{el}$ (-) is the elastic strain tensor, $\varepsilon_{ij}^{pl}$ (-) is the plastic strain tensor, u (m) is the displacement of a particle, and x (m) is the position of the same particle in the deformed configuration.

According to the associate flow rule and the plastic consistency condition:

(38) $$d\varepsilon^{pl}=d\lambda \frac{\partial Q_{p}}{\partial \sigma }=d\lambda \frac{\partial F_{yield}}{\partial \sigma },\;$$

(39) $${\left(\frac{\partial F_{yield}}{\partial \sigma }\right)}^{T}d\sigma +{\left(\frac{\partial F_{yield}}{\partial \varepsilon^{pl}}\right)}^{T}d\varepsilon^{pl}=0,\;$$

where dλ is the harding parameter, Q_p_ is the plastic potential, and F_yield_ is the yield surface.

The yield surface of Hoek and Brown [75], dependent on the unconfined compressive strength (UCS) and tensile strength (T_f_), is used in this study. The Hoek–Brown (HB) yield surface has the form

(40) $$F_{yield}=2\sqrt{J_{2}}sin\left(\theta_{L}+\frac{\pi }{3}\right)-UCS\sqrt{1-m_{HB}\frac{\sigma_{1}}{UCS}},\;$$

(41) $$cos3\theta_{L}=\frac{3\sqrt{3}}{2}\cdot \frac{J_{3}}{J_{2}^{3/2}},\;$$

where θ_L_ (0 ≤ θ_L_ ≤ π/3) is the Lode angle, σ_1_ is the first principal stress, m_HB_ (-) is a material constant parameter which equals 9.9 in this study, and J_2_ and J_3_ are, respectively, the second and third deviatoric stress invariants.

We used the fine content in our previous works [76,77] to show the effect of suction on the unconfined compressive strength (UCS) of unstabilized rammed earth (URE) compacted with the average dry density of 2000 kg·m^−3^, expressed as follows:

(42) $$UCS=\left[u_{ref}+u_{1}\cdot ln\left(s+1\right)\right]\cdot \left(e^{c_{fi}}-1\right),\;$$

where u_ref_ (MPa) is the reference stress and u_1_ (-) is a constant parameter. These parameters are, respectively, considered 1 and 0.829 in this study, and the suction s has the unit of MPa.

Different tensile strength values are also reported by several authors [18,78,79]. In this study, the tensile strength is related to the compressive strength with a coefficient of 0.1, also obtained by Bui et al. [80] and recommended by Meek et al. [81].

The power law [19,82] is used for the elastic modulus at failure (E) with the results reported in the literature [15,18,83,84,85] for various unconfined compressive strength (UCS) and fine content (c_fi_) values:

(43) $$E=E_{ref}\cdot \left(1-c_{fi}\right)\cdot {\left(\frac{UCS}{u_{ref}}\right)}^{e_{1}},\;$$

where E_ref_ (MPa) is the reference elastic modulus at the reference unconfined compressive strength level (u_ref_ = 1 MPa). e_1_ (-) is a constant parameter. E_ref_ and e_1_ are, respectively, equal to 143.2 and 1.766.

All these equations are implemented in the COMSOL finite element software (Version 5.3). The capacity of the proposed theoretical framework to predict thermo-hydro-mechanical (THM) properties of unstabilized rammed earth (URE) materials is studied in our previous works [76,77]. The comparison results show that the proposed numerical framework has a good capacity to predict the THM characteristics of URE materials. After that, the authors extend the numerical simulations of URE constructions.

Before illustrating the simulation results, it should be pointed out that the proposed theoretical framework is only suitable for the RE in France or any place with similar geo-meteorological conditions. As the relative humidity in Luxor (Egypt) drops below 0.3 or the average annual temperature is −4.84 °C in Canada, some of the governing equations and boundary conditions used in this study are not suitable in these places. In addition, the constant parameters in the proposed model are measured by the indoor environment, and they are used to predict the behavior of the URE structure under in situ conditions. With the results from more in situ tests, the accuracy of the proposed numerical framework can be improved. Moreover, chemical reactions [86], crack, freezing, and cyclic thermo-hydro-mechanical (THM) loadings [87] are not considered in this study.

## 3. Numerical Simulations for the Reference Case Study

**Figure 1:**
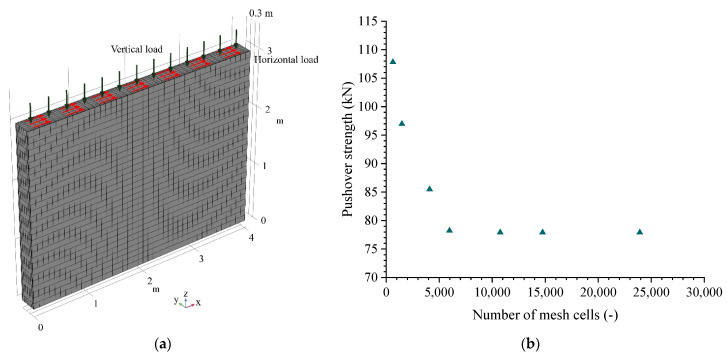
(**a**) Geometry and mechanical boundary conditions of the reference wall. (**b**) Selection of the wall mesh.

First, the long-term (five years [19,88]) behavior of a reference unstabilized rammed earth (URE) wall corresponding to a typical two-story residential building (vertical load of 60 kPa) is initially investigated. It has a dry density of 2000 kg·m^−3^, a fine content of 0.5, a height of 3 m, a thickness of 0.3 m, and a width of 4 m. The following boundary conditions are considered for this reference wall: initial saturation of 0.75, average annual outdoor relative humidity of 0.75, average annual outdoor temperature of 12 °C, ground surface suction of 1 MPa, outdoor wind speed at 10 m height of 3.5 m·s^−1^, cloud cover of 0.35, Munsell color value of 3.5, incoming shortwave radiation of 116 W·m^−2^, and emissivity of 0.96. All these values are summarized in Table 1. The geometry of the reference wall and its mesh are shown in Figure 1a. In addition, the mesh of the wall has a total volume of 3.6 m^3^, and the number of mesh cells is 6000. A mesh independence study is performed to ensure that the numerical results are independent of the grid. Six mesh models with different mesh qualities, namely extremely coarser (650 cells), coarser (1500 cells), coarse (4125 cells), normal mesh (6000 cells), fine mesh (10,800 cells), finer mesh (14,805 cells), and extra fine mesh (24,000 cells), are analyzed, keeping the same conditions. Figure 1b presents a comparison of the pushover strength of the studied wall after 1 day of construction. It can be observed from Figure 1b that when the mesh density is increased to the fourth level, the pushover strength has an insignificant difference by further improving mesh densities. Therefore, the mesh at the fourth level with 6000 cells is used for the following numerical analysis considering the computational accuracy and computation time.

The heat source of radiation and the wind velocity is neglected on the indoor side of the wall, and the indoor relative humidity of the wall (RH_int_) is correlated to the outdoor relative humidity of the wall (RH_ext_) as follows [89]:

(44) $$RH_{int}=0.45RH_{ext}+0.17$$.

The indoor temperature (T_int_) is correlated with the outdoor temperature (T_ext_) as follows [85]:

(45) $$T_{int}=\{\begin{array}{c} 18.9+0.04T_{ext},T_{ext}<12.7{}^{\circ}C\\ 14.201+0.41T_{ext},T_{ext}>12.7{}^{\circ}C \end{array}.\;$$

The movement at the bottom of the wall is fixed while the other displacements are free, and the suction and temperature on the bottom of the wall are equal to the ground surface suction and the average annual outdoor temperature.

To simulate the ultimate condition, the horizontal force on the top surface of the wall is loaded with a loading speed of 1 kN·s^−1^. The simulations are performed for a period of 5 years after the construction of the walls.

**Figure 2:**
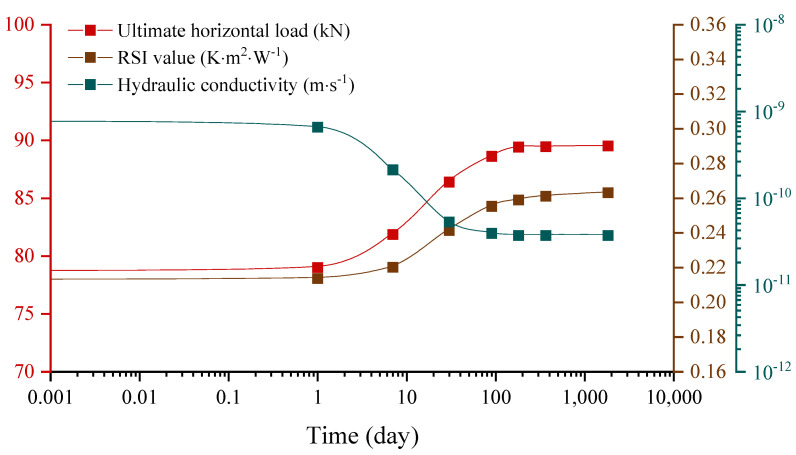
Variations of the ultimate horizontal load, thermal insulation and water permeability of the reference wall within five years.

The main thermo-hydro-mechanical (THM) characteristics of the reference unstabilized rammed earth (URE) wall (in France) are simulated. The average hydraulic conductivity, RSI value, and ultimate horizontal load of the reference wall are used to show the waterproof capacity (hydraulic conductivity), thermal insulation (RSI value), and pushover strength (ultimate horizontal load) of the wall. The simulation results are presented in Figure 2 with the time on a logarithmic scale.

It can be observed from Figure 2 that the hydraulic conductivity, RSI value, and pushover strength of the reference wall decrease by 10%, increase by 24%, and increase by 14%, respectively, after five years. This means that a drying process occurs during construction which is beneficial for the thermo-hydro-mechanical (THM) properties of unstabilized rammed earth (URE) structures. In addition, these THM properties change significantly in the first 100 days and then tend to be stabilized after 180 days.

**Figure 3:**
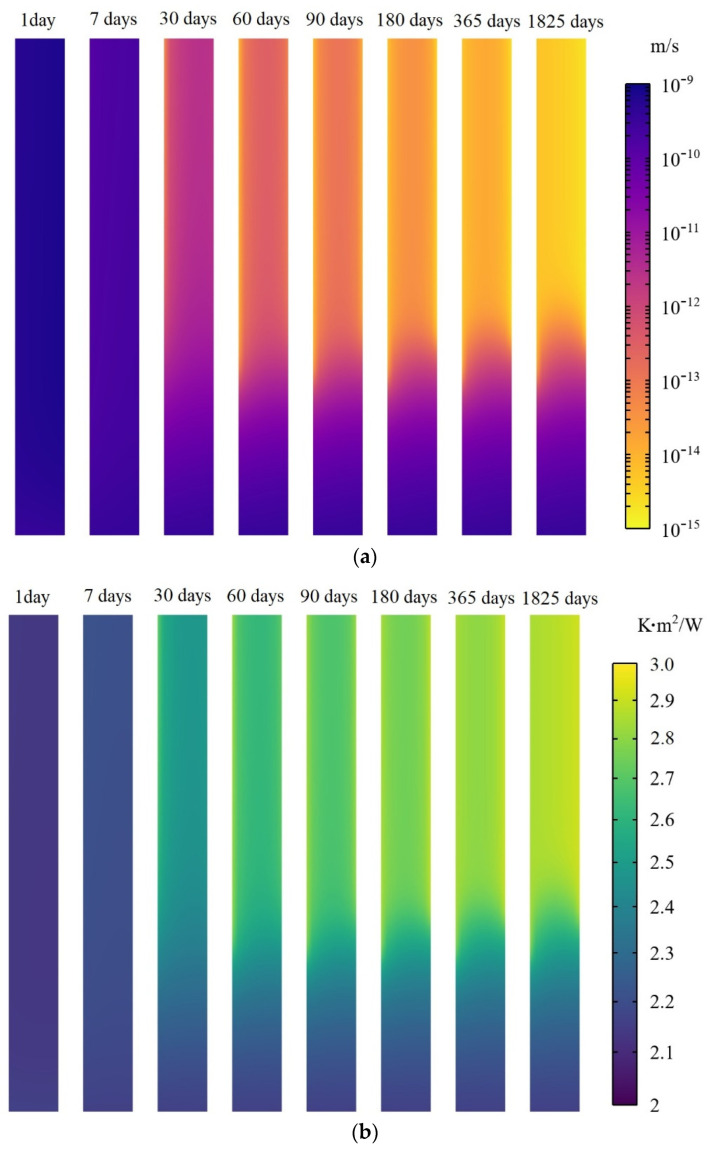
(**a**)**.** Distribution of hydraulic conductivity in the wall according to time. (**b**). Distribution of RSI value in the wall according to time.

The average RSI value and hydraulic conductivity show the thermal insulation and waterproof capacity of the wall in a global view. However, the material properties in the wall do not always remain the same but vary in different areas. In addition, due to the interactions of the environment, the distributions of the material properties in the wall may also change with time. Therefore, a vertical cut plane located in the middle part of the wall is selected to demonstrate the non-homogeneous character of the wall, and eight different times, namely, 1 day, 7 days, 30 days, 60 days, 90 days, 180 days, 365 days, and 1825 days following construction, are used to show the effect of time. The left side of the cut plane represents the interior (indoor), while the right side of the wall represents the exterior (outdoor). The distribution of hydraulic conductivity and RSI value in the wall according to time are illustrated in Figure 3a and Figure 3b, respectively.

It can be observed from Figure 3a,b that the hydraulic conductivity and RSI value in different parts of the wall almost remain the same after 1 day of construction since the water has not had enough time to evaporate through the wall surfaces. In the following days, the water in the wall continuously evaporates through the surfaces of the wall, which results in a decrease in hydraulic conductivity and an increase in RSI value. After 180 days following construction, the distributions of the material properties of the wall gradually become stable and no longer change significantly as time increases. Moreover, the hydraulic conductivity in the upper part of the wall is lower than that of the lower part; the RSI value in the upper part of the wall is higher than that of the lower part. This is mainly caused by the fact that the lower part of the wall is wetter than the upper part due to the effect of rising dampness from underground soil. In addition, indoor air is drier than outdoor air, resulting in lower hydraulic conductivity and a higher RSI value of the wall adjacent to the indoor portion than the outdoor portion.

## 4. Effect of 15 Different Factors on THM Properties of URE Walls

The variation of thermo-hydro-mechanical (THM) properties of the reference wall is detailed in the previous section. In this section, the proposed numerical approach is used to simulate the influence of 15 different factors on THM behaviors of unstabilized rammed earth (URE) walls. Each factor is varied in a conventionally accepted range. The range of variation of each factor is summarized and presented in Table 1. The values taken for the reference wall are also stated in Table 1.

### 4.1. Initial THM Properties

**Figure 4:**
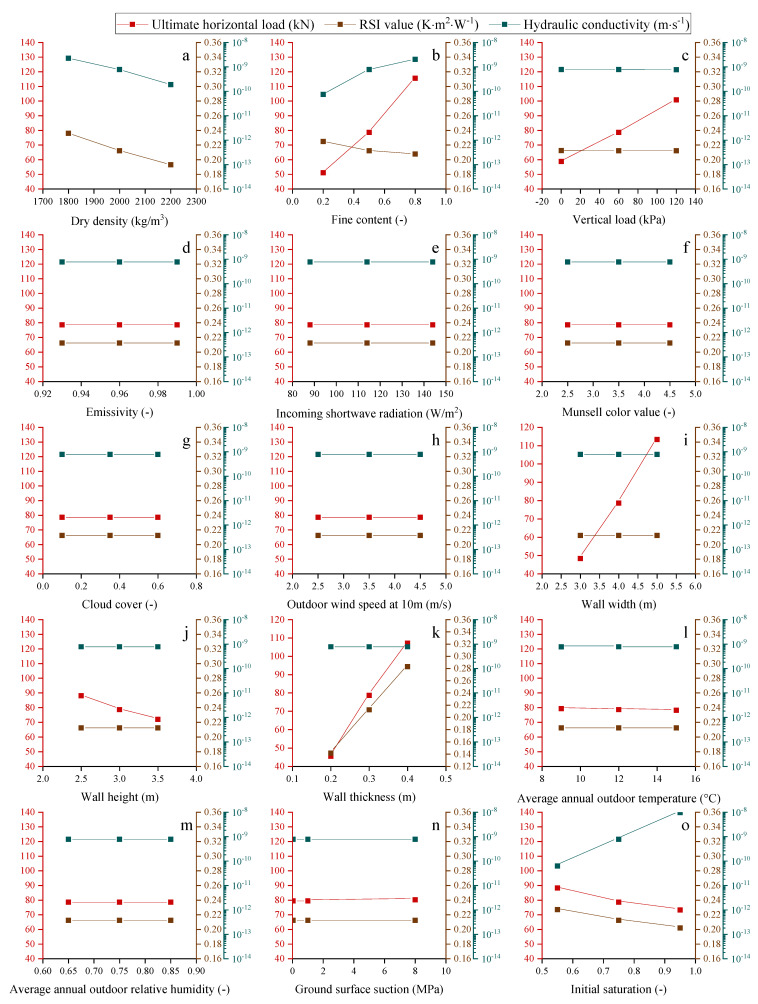
Variations of the average RSI value, hydraulic conductivity, and pushover strength with the influences of different factors for the newly built walls.

Figure 4 shows the initial thermo-hydro-mechanical (THM) properties of unstabilized rammed earth (URE) walls (t = 0) with the impact of 15 different factors. It shows that the average hydraulic conductivity increases with the initial saturation and fine content of the wall, while it decreases when the dry density increases. In addition, the average RSI value increases with the wall thickness and decreases with the dry density, fine content, and initial saturation of the walls. The other factors have negligible effects on the waterproof capacity and thermal insulation of the walls since the interaction between the wall and its surrounding environment has not yet started. Moreover, the ultimate horizontal load increases with fine content, vertical load, wall width, wall thickness, and ground surface suction, while its initial value decreases with wall height and initial saturation.

**Figure 5:**
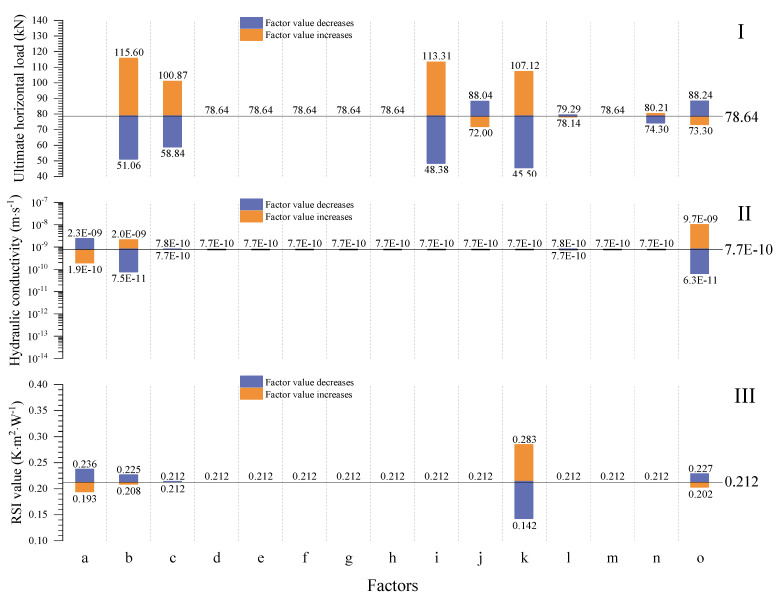
Variations of (**I**) the pushover strength, (**II**) the average hydraulic conductivity, and (**III**) the average RSI value of URE walls according to different factors. (a) Dry density, (b) fine content, (c) vertical load, (d) emissivity, (e) incoming shortwave radiation, (f) Munsell color value, (g) cloud cover, (h) outdoor wind speed at 10 m, (i) wall width, (j) wall height, (k) wall thickness, (l) average annual outdoor temperature, (m) average annual outdoor relative humidity, (n) ground surface suction, and (o) initial saturation of the wall.

Figure 5 shows that the fine content, vertical load, width, length, height, and initial saturation of unstabilized rammed earth (URE) walls greatly influence their initial pushover strength. Perić et al. [90] and Ávila et al. [3] investigated the mechanical behavior of URE through a literature review, and their results show that there is no significant trend between dry density and strength. However, the experimental results from Bruno et al. [91] show that the strength of URE increases as dry density increases. Therefore, the effect of dry density onthe strength needs to be investigated more thoroughly by keeping other influencing factors, for example, water content, fine content, etc., at constant values. For this reason, the effect of dry density on the strength of URE is not investigated in this work. Apart from the effect of dry density, the simulation results show that the fine content is the most crucial factor influencing the initial pushover strength of the studied wall.

From Figure 5, it can also be observed that both the initial waterproof capacity and thermal insulation of URE walls are commonly dominated by the dry density, fine content, and initial saturation. The wall thickness and initial saturation are, respectively, the most significant factors for initial RSI and hydraulic conductivity values.

By following the results presented in Figure 4 and Figure 5, three analytical solutions are proposed to calculate the initial average RSI value (RSI_av-0_), average hydraulic conductivity (K_av-0_), and pushover strength (P_us-0_) of the newly built URE walls:

(46) $$RSI_{av-0}=0.212\cdot \left[\left(1-0.506\cdot \frac{\left(\rho_{d}-2000\right)}{1000}\right)\cdot \left(1-0.136\cdot \left(c_{fi}-0.5\right)\right)\cdot \left(1-0.296\cdot \left(S_{r0}-0.75\right)\right)\right],\;$$

(47) $$K_{av-0}=7.7^{-10}\cdot {\left(1+\left(c_{fi}-0.5\right)\right)}^{4}\cdot {\left(1+\left(S_{r0}-0.75\right)\right)}^{13.88}\cdot {\left(1+\frac{\left(\rho_{d}-2000\right)}{1000}\right)}^{-5},\;$$

(48) $$P_{us-0}=78.64\cdot {\left[\begin{array}{c} \left(1+1.002\cdot \left(c_{fi}-0.5\right)\right)\cdot \left(1+0.003\cdot (P_{v}-60)\right)\cdot \left(1+0.299\cdot \left(w_{id}-4\right)\right)\cdot \\ \left(1-0.148\cdot \left(h_{ei}-3\right)\right)\cdot \left(1+2.798\cdot \left(t_{hi}-0.3\right)\right)\cdot \left(1-0.345\cdot \left(S_{r0}-0.75\right)\right) \end{array}\right]}^{1.38},$$

where S_r0_ (-) is the initial saturation, P_v_ (kPa) is the vertical load, w_id_ (m) is the wall width, h_ei_ (m) is the wall height, and t_hi_ (m) is the wall thickness.

The experimental results from Galaa et al. [92] are used to verify the ability of the proposed approach to predict the hydraulic conductivity of an unstabilized rammed earth (URE) sample. Equation (48) is used for calculation since the cylinder sample was in the saturated condition (initial saturated at t = 0 days). Comparison (with an error of 7% for the studied case) of the measured and predicted hydraulic conductivity is presented in Table 2.

### 4.2. Stabilized THM Properties after 5 Years

**Figure 6:**
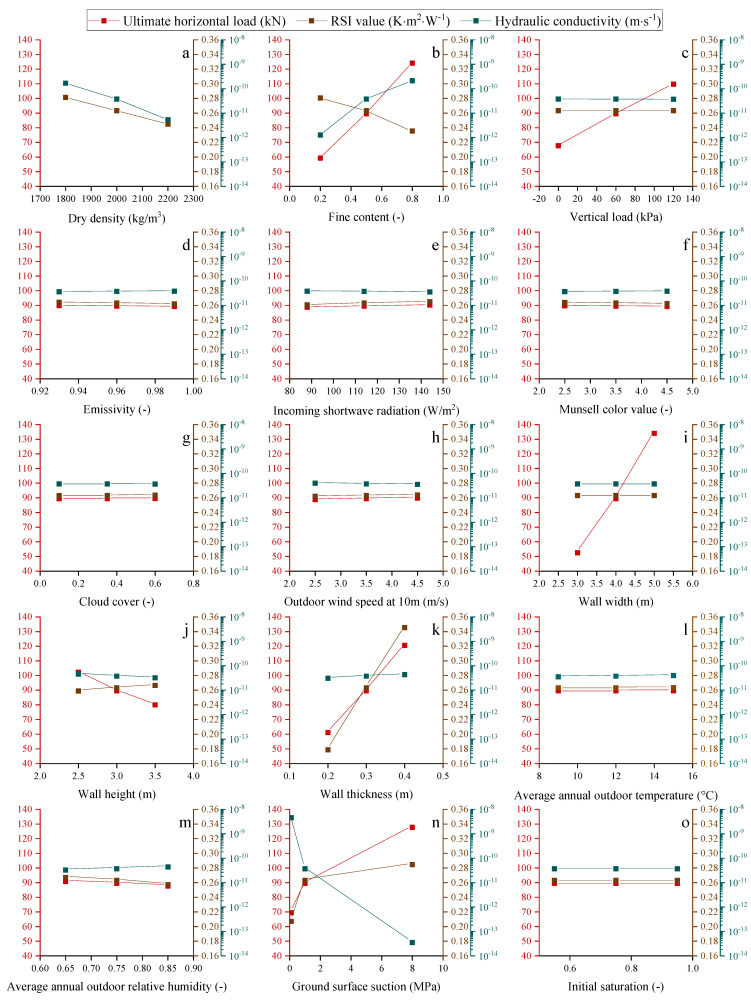
Variations of the average RSI value, hydraulic conductivity, and pushover strength with the influences of different factors for the walls built after five years.

As shown in Figure 6, the thermo-hydro-mechanical (THM) properties of the walls constructed after five years are also investigated to determine their final performance in relation to various factors.

The average hydraulic conductivity increases with wall thickness and outdoor relative humidity, as shown in Figure 6. In addition, fine content increases the average hydraulic conductivity of the wall. This is due to the fact that walls with a higher fine content have smaller pores than those with a lower fine content, resulting in a reduced capillary radius. The average hydraulic conductivity of the wall decreases as the wall height rises because the wall height rise enlarges the dryer parts above the balanced height. Increased ground surface suction reduces the rising damp height and the wall’s average hydraulic conductivity. Increased dry density decreases the wall’s porosity, resulting in decreased water permeability and average hydraulic conductivity.

Furthermore, the vertical load, emissivity, incoming shortwave radiation, Munsell color value, cloud cover, outdoor wind speed at 10 m height, wall width, average annual outdoor temperature, and initial saturation have a minor effect on the average hydraulic conductivity of the wall.

The increase in height dries the wall; thus, its average thermal conductivity decreases or its average RSI value increases when the wall height increases. In addition, increasing the wall’s thickness improves its thermal insulation. The average RSI value decreases as the average annual outdoor relative humidity and fine content both increase the amount of water stored in the walls, assuming all other boundary conditions remain unchanged. Greater ground suction increases the average RSI of the wall. In addition, the number of soil particles increases as dry density rises, resulting in a lower average RSI value for the wall.

The vertical load, emissivity, incoming shortwave radiation, Munsell color value, cloud cover, outdoor wind speed at 10 m height, wall width, average annual outdoor temperature, and initial saturation have relatively little effect on the thermal insulation capacity of the walls.

The simulation results show that wall strength increases with the increase in wall thickness, and the increase in the average annual outdoor relative humidity decreases the shear strength of the wall. On the contrary, the increase in ground surface suction increases the strength of the wall. The wall pushover strength also increases as the fine content increases, probably caused by the binder effects of fine content.

**Figure 7:**
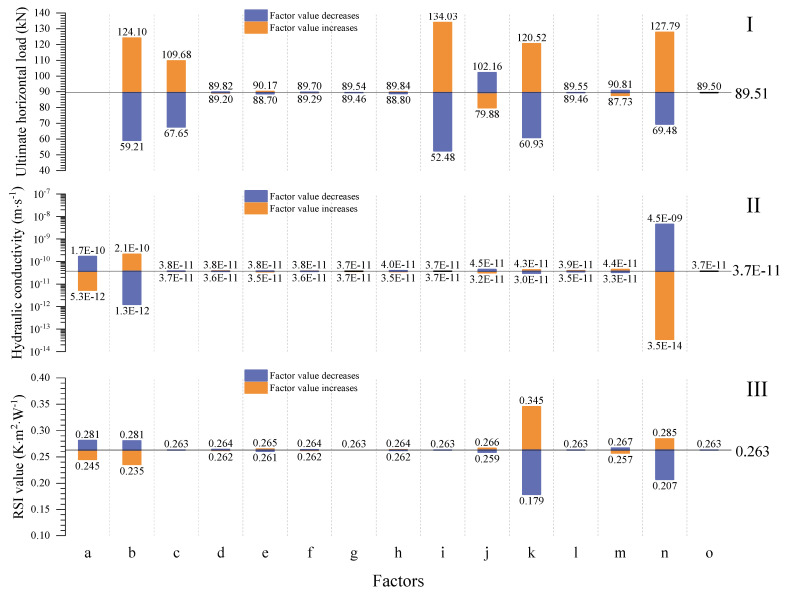
Variations of the (**I**) pushover strength, (**II**) average hydraulic conductivity, and (**III**) average RSI value of the walls built after five years according to (a) dry density, (b) fine content, (c) vertical load, (d) emissivity, (e) incoming shortwave radiation, (f) Munsell color value, (g) cloud cover, (h) outdoor wind speed at 10 m, (i) wall width, (j) wall height, (k) wall thickness, (l) average annual outdoor temperature, (m) average annual outdoor relative humidity, (n) ground surface suction, and (o) initial saturation of the wall.

The influence of the above 15 factors on the average hydraulic conductivity, RSI value, and ultimate horizontal load of the investigated walls after five years is summarized in Figure 7.

The results in Figure 7 show that 5 years after construction, the waterproof capacity of the walls is primarily influenced by the soil surface suction, fine content, and dry density. Among these factors, the simulation results reveal that ground surface suction is the most crucial factor that affects the waterproof capacity of the walls. The waterproof capacity of the wall can be improved by reducing the fine content value, raising the damp effect, and increasing the dry density of the wall. The thermal insulation characteristics of the wall are dominated by the wall thickness, soil surface suction, fine content, and dry density. Moreover, the simulation results show that wall thickness is the most crucial factor influencing the thermal insulation of the walls. The thermal insulation properties of the wall can be improved by increasing the wall thickness and reducing the rising dampness, fine content, and dry density. In addition, the results in Figure 7 show that the pushover strength of the wall is greatly affected by the wall width, fine content, wall thickness, soil surface suction, vertical load, and wall height. Additionally, wall width is considered to be the most influencing factor according to the simulation results. The pushover strength of the wall can be improved by increasing the wall width, fine content, wall thickness, and vertical load, and decreasing the rising dampness and wall height.

Based on the simulation results presented in Figure 6 and Figure 7, three analytical solutions are proposed for the stabilized average RSI value (RSI_av-1825_), average hydraulic conductivity (K_av-1825_), and pushover strength (P_us-1825_) of the URE walls built after five years:

(49) $$RSI_{av-1825}=0.263\cdot \left[\begin{array}{c} \left(1-0.345\cdot \frac{\left(\rho_{d}-2000\right)}{1000}\right)\cdot \left(1-0.287\cdot \left(c_{fi}-0.5\right)\right)\\ \cdot \left(1+3.169\cdot \left(t_{hi}-0.3\right)\right)\cdot {\left(1+\left(s_{g}-1\right)\right)}^{0.065} \end{array}\right],\;$$

(50) $$K_{av-1825}=3.7^{-11}\cdot {\left(1+\frac{\left(\rho_{d}-2000\right)}{1000}\right)}^{-5}\cdot {\left(1+\left(c_{fi}-0.5\right)\right)}^{4}\cdot {\left(1+\left(s_{g}-1\right)\right)}^{-2.08},\;$$

(51) $$P_{us-1825}=89.51\cdot {\left[\begin{array}{c} \left(1+0.236\cdot \frac{\left(\rho_{d}-2000\right)}{1000}\right)\cdot \left(1+0.797\cdot \left(c_{fi}-0.5\right)\right)\cdot \left(1+0.003\cdot (P_{v}-60)\right)\\ \cdot \left(1+0.301\cdot \left(w_{id}-4\right)\right)\cdot \left(1-0.165\cdot \left(h_{ei}-3\right)\right)\cdot \left(1+2.189\cdot \left(t_{hi}-0.3\right)\right)\cdot {\left(1+\left(s_{g}-1\right)\right)}^{0.103} \end{array}\right]}^{1.521},\;$$

where the s_g_ (MPa) is the suction of the ground soil.

The experimental results from Jiang et al. [17] are used to evaluate the approach capacity to predict the RSI value. Since the RSI value in the thickness direction is obtained under equilibrium conditions in the laboratory (corresponding to 1825 days), Equation (49) is used to predict its RSI value. The comparison of the measured and predicted RSI values is presented in Table 3.

Globally, dry density, vertical load, fine content, wall dimensions, and rising dampness have a significant effect on the thermo-hydro-mechanical (THM) properties of unstabilized rammed earth (URE) walls. A suitable material texture, compaction condition, wall dimension, and technique reducing the rising dampness are required during the design stage of the structures. For existing structures, the wall dimension, material type, and loading state are already fixed. Therefore, any strategy preventing the rising dampness is recommended for URE structures to improve their THM characteristics.

## 5. General THM Long-Term Behaviors of a URE

**Figure 8:**
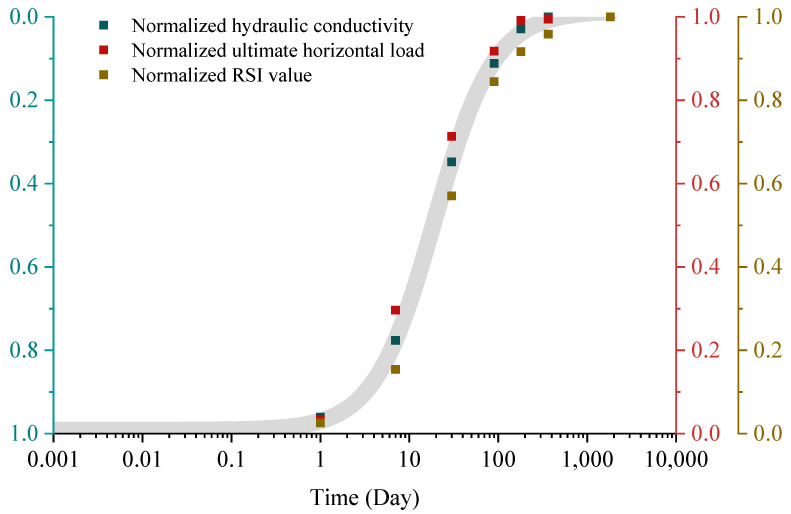
Normalized THM values for the reference wall.

As presented in Figure 2, the thermo-hydro-mechanical (THM) properties of the unstabilized rammed earth (URE) wall change gradually with time. By normalizing the curves in Figure 2, we notice that the normalized curves have similar shapes as presented in Figure 8. It means that time has a similar influence on the average RSI value, average hydraulic conductivity, and pushover strength of the wall. This indicates that the hydraulic, thermal, and mechanical properties of the wall are interconnected, and any parameter can be adopted to predict the other two. This intrinsic connection provides the possibility to predict, for example, the material strength through water content as studied by Vásárhelyi and Ván [93] or the soil thermal conductivity based on its penetration resistance and water content as presented by Usowicz et al. [94]. Thus, the average hydraulic conductivity is selected due to its fewer interference factors (Figure 5 and Figure 7) with only three influencing factors (wall thickness, dry density, and fine content).

The same normalization procedure is conducted for different dry densities, ground soil suctions, and fine contents. After that, the normalized curves are calibrated using the following equation:

(52) $$N_{or}=\frac{1}{1+{\left(\frac{t}{t_{or}}\right)}^{1.5}},\;$$

where N_or_ (-) is the normalized parameter, and t_or_ (day) is the normalized reference time parameter. Since t_or_ is mainly dependent on the dry density ρ_d_, the fine content c_fi_, and the ground soil suction s, Table 4 illustrates the variation of t_or_ with these parameters.

**Figure 9:**
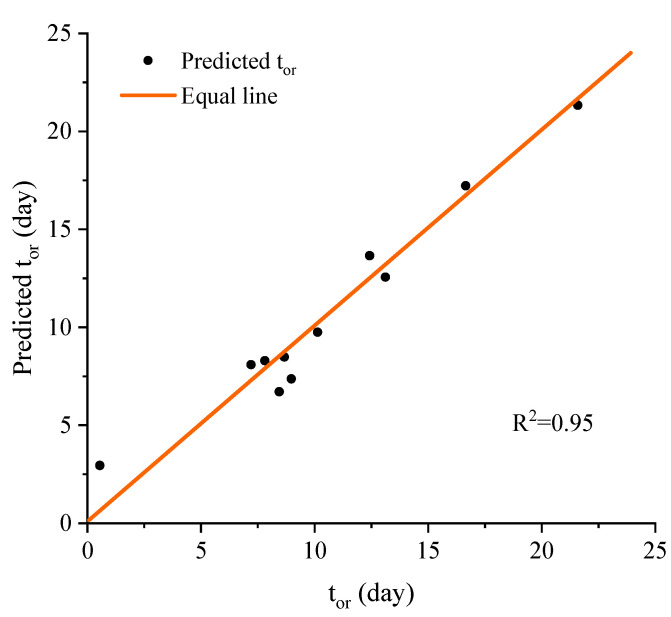
Comparations of tor and the predicted tor through Equation (53).

The parameter t_no_ is then correlated to the ground soil suction, fine content, and dry density through the following equation (Figure 9):

(53) $$t_{or}=s_{g}{}^{0.457}\cdot c_{fi}{}^{-0.456}\cdot \rho_{d}{}^{0.236}.\;$$

Eventually, the effect of time on the pushover strength of the unstabilized rammed earth (URE) wall can be obtained with the following equation:

(54) $$P_{us}=\{\begin{array}{c} N_{or}\cdot \left(P_{us-0}-P_{us-1825}\right)+P_{us-1825},\;P_{us-0}>P_{us-1825}\\ \left(1-N_{or}\right)\cdot \left(P_{us-1825}-P_{us-0}\right)+P_{us-0},\;P_{us-0}<P_{us-1825} \end{array},\;$$

where P_us_ is the related pushover strength of unstabilized rammed earth (URE) wall, and P_us-0_ and P_us-1825_ represent the pushover strength of newly built and five years aging URE walls, respectively.

In addition, the average RSI value of the unstabilized rammed earth (URE) wall can be calculated using time as follows:

(55) $$RSI_{av}=\{\begin{array}{c} N_{or}\cdot \left(RSI_{av-0}-RSI_{av-1825}\right)+RSI_{av-1825},\;RSI_{av-0}>RSI_{av-1825}\\ \left(1-N_{or}\right)\cdot \left(RSI_{av-1825}-RSI_{av-0}\right)+RSI_{av-0},\;RSI_{av-0}<RSI_{av-1825} \end{array},\;$$

where RSI_av_ is the related RSI value of the unstabilized rammed earth (URE) wall, and RSI_av-0_ and RSI_av-1825_ are, respectively, the average RSI value of newly built and five years aging URE wall.

The average hydraulic conductivity of the wall is presented as

(56) $$K_{av}=\{\begin{array}{c} EXP\left[N_{or}\cdot \left(lnK_{av-0}-lnK_{av-1825}\right)+lnK_{av-1825}\right],\;K_{av-0}>K_{av-1825}\\ EXP\left[\left(1-N_{or}\right)\cdot \left(lnK_{av-1825}-lnK_{av-0}\right)+lnK_{av-0}\right],\;K_{av-0}<K_{av-1825} \end{array},$$

where K_av_ is the related hydraulic conductivity of the unstabilized rammed earth (URE) wall, and K_av-0_ and K_av-1825_ represent the average hydraulic conductivity of newly built and five years aging URE walls, respectively.

The proposed approach is used to predict the pushover strength values of unstabilized rammed earth (URE) walls reported by El Nabouch [28]. The walls are dried for two months with an ambient air temperature of 20℃ and relative humidity of 0.6. After that, the pushover tests are carried out. The wall dimensions, material type parameters, ambient air conditions, vertical loads, measured pushover strengths, and predicted pushover strength are summarized in Table 5.

**Figure 10:**
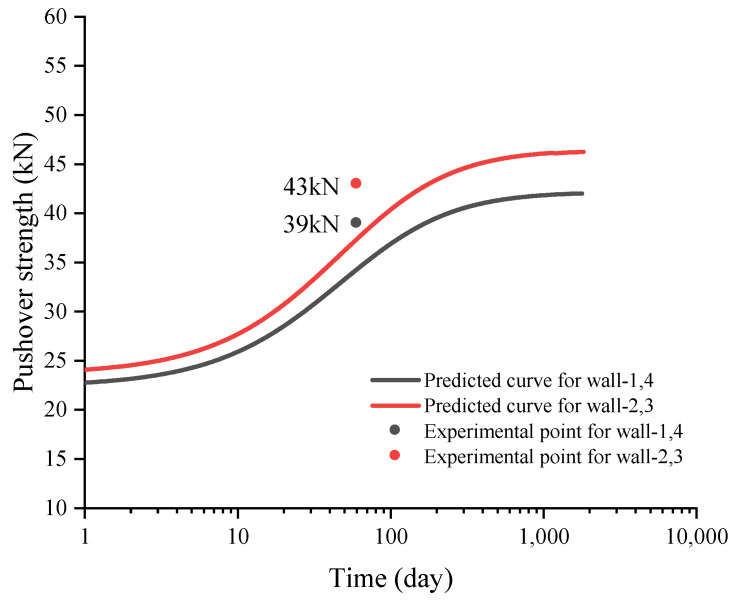
Comparison between the predicted and experimental results about the pushover strength.

Using the analytical model in Section 4, the long-term pushover strength of the walls can be predicted from the initial stage where the walls are constructed. The comparison between the predictions and the experimental results is presented in Figure 10.

**Figure 11:**
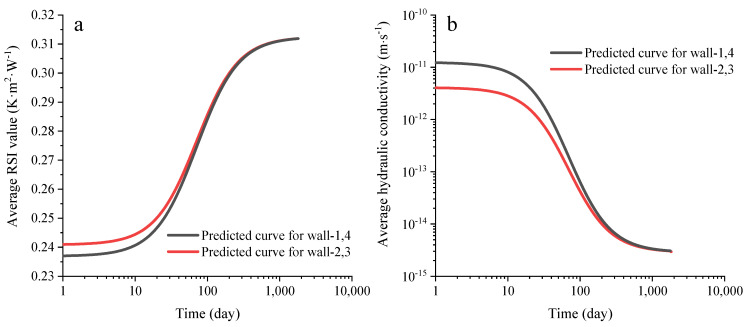
Predicted (**a**) average RSI and (**b**) hydraulic conductivity of the RE walls from El Nabouch [28].

In addition, to better understand the thermo-hydro-mechanical (THM) behaviors of the unstabilized rammed earth (URE) wall with the changing of time, the variations of the average RSI value and hydraulic conductivity of the wall are also predicted, and the results are presented in Figure 11a,b.

Globally, the proposed analytical approach provides an easy option to predict the initial or final thermo-hydro-mechanical (THM) properties of unstabilized rammed earth (URE) walls at the design stage. Moreover, this approach provides the possibility to predict the periodical variation of THM properties of URE structures.

It should be pointed out that the proposed analytical equations are established based on limited experimental data. More experimental results are still required for model calibrations and validations.

## 6. Conclusions

In this study, the effect of time was investigated for a typical unstabilized rammed earth (URE) wall from its initial construction stage until five years after its utilization. The simulation results show that time has a positive effect on the waterproof capacity, thermal insulation, and mechanical strength of the URE wall. In addition, these thermo-hydro-mechanical (THM) properties of the URE wall change significantly in the first 100 days and then tend to be stabilized after 180 days for a typical URE wall. Therefore, the application of a drying process before utilization is beneficial to the THM properties of URE structures.

The effect of 15 different factors: fine content, dry density, initial saturation of the wall, ground surface suction, average annual outdoor relative humidity, average annual outdoor temperature, wall thickness, wall height, wall width, outdoor wind speed, cloud cover, Munsell color value, incoming shortwave radiation, emissivity, vertical load on the waterproof capacity, thermal insulation, and pushover strength of unstabilized rammed earth (URE) walls, was investigated in this study.

The simulation results show that the waterproof capacity of the wall can be improved by reducing the fine content value, raising damp effects, and increasing the dry density of the wall. The thermal insulation characteristics of the wall can be improved by increasing the wall thickness and reducing the rising dampness, fine content, and dry density. The pushover strength of the wall can be improved by increasing the wall width, fine content, wall thickness, and vertical load and decreasing the rising damp effects and wall height. It should be noted that the effect of dry density on the pushover strength of the wall is not studied in this work.

Finally, a new approach was proposed to predict the thermo-hydro-mechanical (THM) behaviors of unstabilized rammed earth (URE) walls. The proposed approach was validated by some existing experimental results in the literature. Globally, the proposed analytical approach provides an easy framework to predict the short and long-term THM properties of URE walls. These findings are essential for the designing of URE structures. It provides a global view of the crucial factors affecting URE structures and also offers a possible way to predict THM properties over time. Since the current investigation is based on numerical simulations, further experimental measurements, especially in-situ experimental tests, are still necessary to complete this work.

## Figures and Tables

**Table 1:** Factors studied in this work with their reference values and the range of variation of each factor.

	Factors	Unit	Reference Value	Decrease to	Increase to
Material	fine content	-	0.5	0.2	0.8
	dry density	kg·m^−3^	2000	1800	2200
Hydro-thermal	initial saturation of the wall	-	0.75	0.55	0.95
	ground surface suction	MPa	1	0.1	8
	average annual outdoor relative humidity	-	0.75	0.65	0.85
	average annual outdoor temperature	℃	12	9	15
	outdoor wind speed at 10 m	m·s^−1^	3.5	2.5	4.5
	cloud cover	-	0.35	0.1	0.6
	Munsell color value	-	3.5	2.5	4.5
	incoming shortwave radiation	W·m^−2^	116	88	144
	Emissivity	-	0.96	0.93	0.99
Dimension	wall thickness	m	0.3	0.2	0.4
	wall height	m	3	2.5	3.5
	wall width	m	4	3	5
Mechanical	vertical load	kPa	60	0	120

**Table 2:** Comparison of the measured and predicted hydraulic conductivity.

Dry Density (kg/m^3^)	Fine Content	Temperature (°C)	Suction (MPa)	Measured Hydraulic Conductivity (m·s^−1^)	Predicted Hydraulic Conductivity (m·s^−1^)
1980	0.3	25	0	7.2 × 10^−9^	7.7 × 10^−9^

**Table 3:** Comparison of the measured and predicted RSI value.

	Height (m)	Width (m)	Thickness (m)	Dry Density (kg·m^−3^)	Fine Content (-)	Temperature (°C)
Earth block	0.03	0.05	0.05	2160	0.8	25
	Relative humidity (-)	Suction (MPa)	Vertical load (kPa)	Measured RSI value (K·m^2^·W^−1^)	Predicted RSI value (K·m^2^·W^−1^)	
	0.6	69	0	0.076	0.070	

**Table 4:** Variations of t_or_ according to the dry density, fine content, and ground soil suction.

s (MPa)	t_or_ (-)	ρ_d_ (kg·m^−3^)	t_or_ (-)	c_fi_ (-)	t_or_ (-)
0.1	0.558	1800	7.23	0.2	13.17
1	7.84	2000	7.84	0.5	7.84
8	21.66	2200	8.70	0.8	8.48

**Table 5:** Comparison of the measured and predicted pushover strengths of URE walls.

	Height (m)	Width (m)	Thickness (m)	Dry Density (kg/m^3^)	Fine Content (-)	Temperature (°C)
wall-1,4	1.5	1.5	0.25	1960	0.3	20
wall-2,3	1.5	1.5	0.25	1960	0.3	20
	**Relative Humidity**	**Suction (MPa)**	**Vertical Load (kPa)**	**Initial Saturation (-)**	**Measured Pushover Strength (kN)**	**Predicted Pushover Strength (kN)**
wall-1,4	0.6	69	300	0.53	39	34
wall-2,3	0.6	69	300	0.47	43	37

## Data Availability

Not applicable.
